# Periosteum Organoid: Biomimetic Design Inspired From the Bone Healing Process

**DOI:** 10.1002/EXP.20240298

**Published:** 2025-12-07

**Authors:** Shuyue Hao, Fuxiao Wang, Jingtao Huang, Zhidao Xia, James T. Triffitt, Chenjie Xu, Long Bai, Jiacan Su

**Affiliations:** ^1^ Organoid Research Center Institute of Translational Medicine Shanghai University Shanghai China; ^2^ School of Medicine Shanghai University Shanghai China; ^3^ School of Life Science and Engineering Southwest Jiaotong University Chengdu China; ^4^ School of Materials Science and Engineering Southwest Jiaotong University Chengdu China; ^5^ Department of Orthopedics Shanghai Zhongye Hospital Shanghai China; ^6^ Centre For Nanohealth ILS2 Medical School Swansea University Swansea UK; ^7^ Nuffield Department of Orthopaedics Rheumatology and Musculoskeletal Sciences The Oxford University Institute of Musculoskeletal Sciences The Botnar Research Centre Nuffield Orthopaedic Centre Oxford UK; ^8^ Department of Biomedical Engineering City University of Hong Kong Hong Kong SAR China; ^9^ National Center for Translational Medicine (Shanghai) SHU Branch Shanghai University Shanghai China; ^10^ Wenzhou Institute of Shanghai University Wenzhou China; ^11^ Department of Orthopedics Xinhua Hospital Affiliated to Shanghai Jiao Tong University School of Medicine Shanghai China

**Keywords:** Angiogenesis, Bone Healing, Immunomodulation, Neuromodulation, Periosteal Biomaterials, Periosteum Organoid

## Abstract

Large bone defects present major problems in plastic, maxillofacial, and orthopedic reconstructive surgery. With respect to osseous tissues, currently, autologous and allogeneic bone grafts are commonly used clinical treatments, but there are limitations in terms of donor availability, morbidity, and risk of immunogenic reactions. Tissue‐engineered bone constructs offer promising alternatives but struggle to replicate the complex biological functions of native bone, leading to suboptimal outcomes. The periosteum has been shown to be a key factor in bone regeneration and has a bilayered structure that is essential for bone integrity and repair. However, large bone defects cause damage to the periosteum and weaken its regenerative capacity. Therefore, periosteum organoids have been developed with the help of new organoid technology to achieve accelerated bone regeneration. This technology incorporates a variety of natural/synthetic materials and biologically derived factors that can be endowed with key biological functions for bone regeneration, such as, antimicrobial, immunomodulatory, neuromodulatory, angiogenic, and osteogenic capabilities. This review explores the structure and function of periosteum, the design and application of periosteum organoids and their potential integration with bone organoids. In addition, the recent advances and future directions for the use of such organoids in novel regenerative medicine and bone repair strategies are highlighted.

## Graphical abstract

Periosteum is a key factor in bone regeneration and repair, and the development of periosteum organoids with the help of organoid technology can achieve accelerated bone regeneration. This review explores the structure and function of the periosteum, the design and application of periosteum organoids, and their potential integration with bone organoids, and also highlights recent advances and future directions in the use of such organoids in novel regenerative medicine and bone repair strategies.

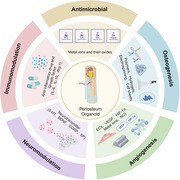

## 1 Introduction

The management of large bone defects, caused by trauma, tumor resection, or congenital anomalies, poses formidable challenges to orthopedic and maxillofacial surgeons [1]. A solution was needed that would meet the complexity of the clinical situation while restoring the functional integrity and biomechanical properties of the affected bone [2, 3]. The gold standard, autologous bone grafting, despite its widespread use, is constrained by donor site morbidity and limited graft availability [4]. Allogeneic bone grafts, while mitigating some of these issues, introduce risks of immunogenic reactions and disease transmission [5, 6]. The development of tissue‐engineered bone has been claimed to significantly accelerate the speed and quality of bone repair. However, this also has severe limitations in fully replicating the biological and functional complexities of native bone tissue, often resulting in less than optimal osseointegration and regeneration outcomes [7, 8, 9]. To address this issue and to reproduce self‐assembling and self‐renewing organs in vitro, the use of three dimensional (3D) culture systems to guide stem cells to form organoids is being proposed [10].

The periosteum, a dual‐layered connective tissue encasing most bones, is intricately structured with an outer fibrous layer, housing longitudinally oriented cells and collagen fibers, and an inner cambial layer, housing multipotent osteogenic stem cells, the so‐called mesenchymal stem cell (MSCs), and derived osteogenic progenitors [11]. This specialized structure not only regulates cellular and collagen alignment but is pivotal in bone integrity, modeling, and remodeling, especially during development and bone defect repair processes [12]. Recent studies have shown that the periosteum has immunomodulatory, neuromodulatory, angiogenic, and osteogenic functions that provide protection, a stable environment, and nutritional support for damaged bone to ensure effective bone repair [13]. Bacterial infection is always a difficult and urgent problem in the treatment of open trauma. Therefore, in addition to strengthening the natural repair capacity of the periosteum, special attention and enhancement of its antimicrobial functions are needed to ensure efficient and comprehensive bone repair. The integrity of the periosteum is necessary for the effective formation of new bone at the periosteal site, and when the periosteum is injured or damaged, it significantly hinders the outcome of bone healing [14]. As a result of the periosteum's significant contribution to bone repair, large bone defects often result in substantial periosteal damage, rendering the natural regenerative capabilities of the periosteum insufficient [15]. The emergence of periosteum organoid technologies provides new ideas to solve these challenges. The emergence of periosteum organoid technology provides an innovative way to address the challenges of bone regeneration and repair. By mimicking the structure and function of the natural periosteum, this technology constructs in vitro periosteum organoids with a high degree of physiological similarity, which are capable of reproducing the complex 3D structural organization and natural functional properties of the periosteum. This process enables precise regulation of the physiological activities of stem cells on a 3D scaffold and mimics biochemical signals and mechanical stimuli in the periosteal microenvironment in vivo. The periosteum organoid can provide a stable scaffold at the damaged site to guide cell growth and differentiation, thus promoting effective new bone formation.

The name “organoid” refers to an organ‐like structure and is derived from the combination of the Greek suffix “oid” and the word “organ,” [16]. Organoids are self‐organizing 3D tissues, usually derived from stem cells, that attempt to mimic the physiological functions, macroscopic/microstructural, and biological complexity of specific organs [17]. Compared with traditional two‐dimensional cell culture, organoid technology can more comprehensively capture the dynamic interactions between cells and between cells and matrix, providing a more accurate and reliable model for drug efficacy assessment, safety prediction, and disease mechanism research [18]. Organoid technology can be used to construct periosteum organoid models with complex physiological functions, providing an important reference for the design and optimization of artificial periosteum. Physiologically, designing a periosteum organoid structure that matches the natural process of bone healing may be a highly efficient repair strategy. Bone healing is a finely tuned process involving multiple overlapping and alternating functional events such as antimicrobial, immunomodulation, neuromodulation, angiogenesis, and osteogenesis [19]. In closed fractures, immune modulation occurs through the primary stages of bone healing, to be followed by neurotrophins secreted by the nervous system and nutrients supplied by neovascularization at the defect site, which synergistically regulate the osteogenic microenvironment [20, 21]. In open fractures the antimicrobial effects of recruited neutrophils to the fracture site supply the first innate defense against infection. Eventually, osteoblasts deposit new osseous tissue that eventually forms mature bone tissue [22]. Innovative strategies have led to the creation of periosteum organoids that employ various materials ranging from native tissues [23], scaffold‐free cell sheets [24], to scaffold‐cell composites [25]. Gao et al. demonstrated that hBMSC cell sheets have excellent osteogenic potential [26]. Liu et al. combined hMSC cell sheets with hydrogel scaffolds, which also demonstrated that this periosteum‐like structure is highly useful for remodeling the bone microenvironment [27]. These periosteum organoids are designed to mimic the natural periosteum's regenerative capabilities for the bone healing process, as aforementioned, thereby potentially enhancing bone regeneration in defects where the natural function of the periosteum is impaired. The periosteum organoid is an example of the mutual integration of biomaterial technology and organoid technology. It not only provides a new way to solve clinical problems, such as the repair of bone defects, but also opens up a new path for the development of regenerative medicine. With the continuous progress and innovation of technology, it is reasonable to believe that periosteum organoids will play an even more important role in the future in the medical field.

**FIGURE 1:**
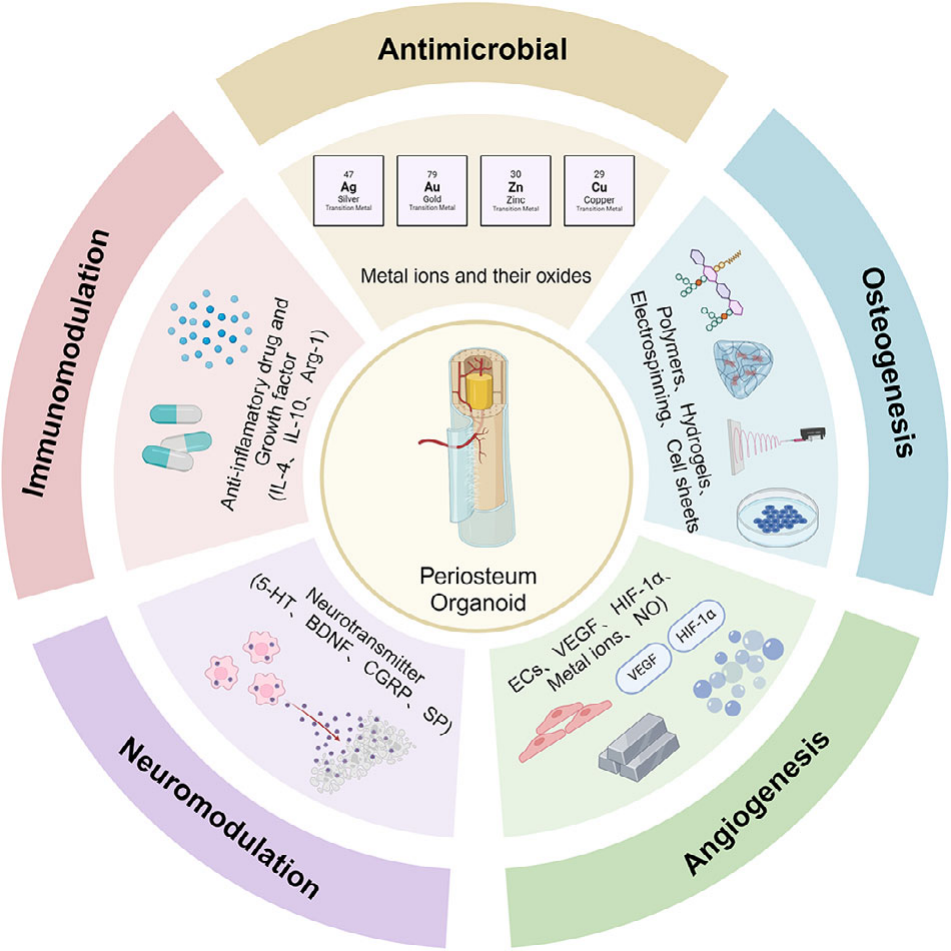
Strategies for constructing periosteum organoid. Created with BioRender.com.

In this comprehensive review, we embark on an exploration of the current knowledge of the periosteum, beginning with an intricate delineation of the architecture and its pivotal roles in bone physiology. Recognizing the periosteum's multifaceted functions provides a fundamental framework for the conceptualization and development of periosteum organoids. Delving deeper, we investigate the complex engineering approach in tissue engineering and detail the application of different functional periosteum organoids described for bone repair (Figure 1). Furthermore, we delineate prospective trajectories in the integration of periosteum organoids with bone organoids, spotlighting the potential for significant advancements in therapeutic applications within clinical settings. By bridging the gap between theoretical insights and practical applications, this review aims to underscore the transformative implications of the synergic use of periosteum organoids and bone organoids in regenerative medicine and for bone repair strategies.

## 2 History of Organoid Development

With the rapid development of regenerative medicine, organoid technology, with its unique “human modeling” system, brings a revolutionary new approach to the fields of human disease modeling, tissue engineering, drug discovery, and diagnostics, and shows great potential for development [28]. Organoids, derived from primary tissues or differentiated from pluripotent stem cells, possess structural and functional properties similar to those of natural organs, including the ability to self‐assemble and self‐renew [29, 30]. Compared to the traditional 2D monolayer cell culture model, 3D cell culture models allow cells to grow in multiple dimensions under in vitro conditions, thus more accurately mimicking the complex environment in vivo. This high degree of mimicry of in vivo conditions allows organoids to be stable in extended culture systems, providing unprecedented opportunities for medical research and clinical applications.

**FIGURE 2:**
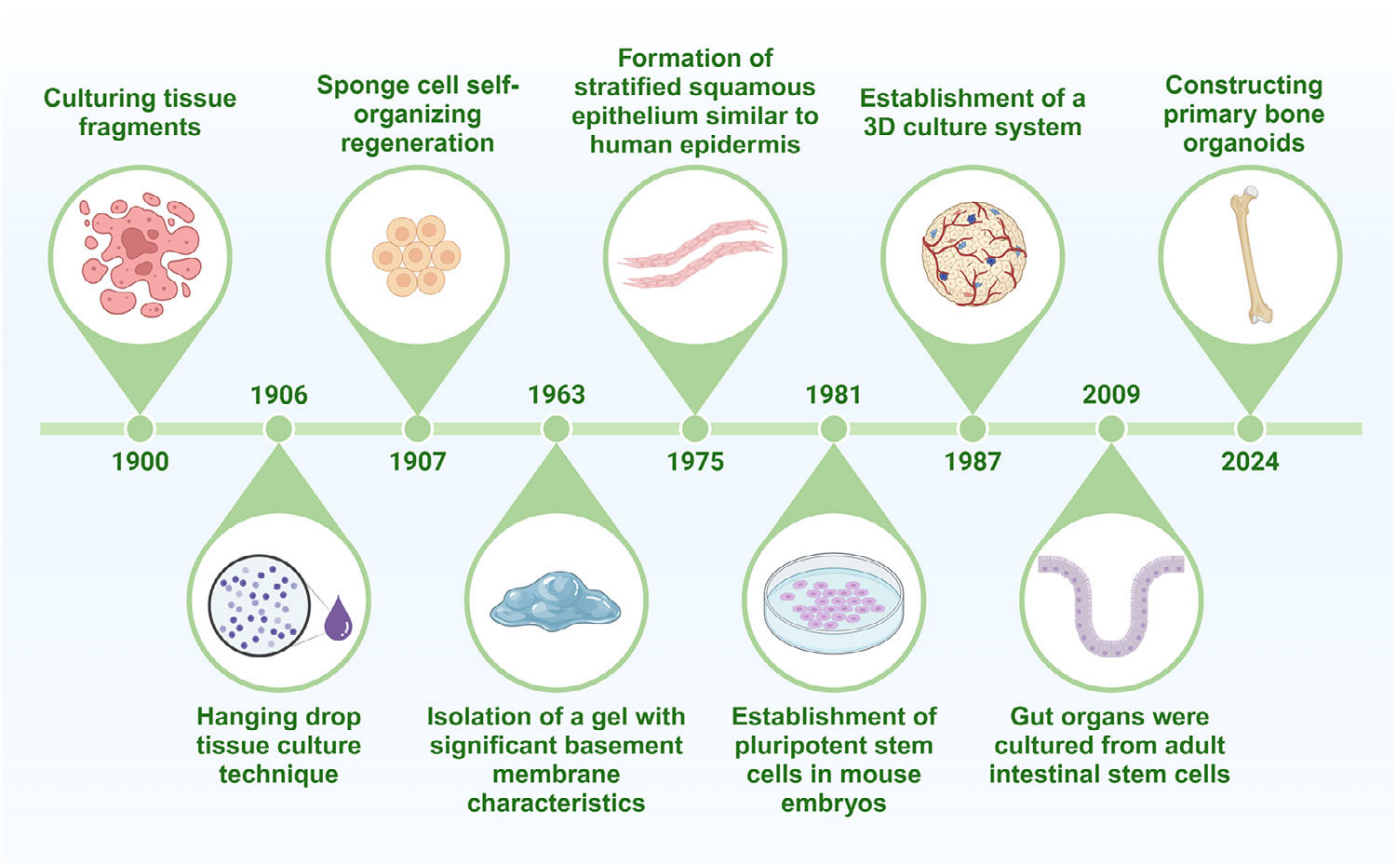
History of organoid development. Created with BioRender.com.

Organoid technology has been gradually established and continuously developed since the beginning of the last century (Figure 2). As early as 1900, researchers wanted to reproduce organ formation in cultures, and they tried to start by culturing tissue fragments. Harrison developed the hanging drop tissue culture technique in 1906 [31]. In 1907, Wilson et al. first verified the ability of isolated sponge cells to self‐organize and regenerate whole organisms, which laid the foundation for subsequent organoid research [32]. In 1963, Swarm and his team explored in depth the properties of the extracellular matrix of what was thought to be a chondrosarcoma, and in the process succeeded in isolating a gel with remarkable basement membrane characteristics [33]. This extracellular matrix gel was named EHS sarcoma gel because of its unique properties, and it later became commercially available and a widely used matrix gel in organoid technology. This discovery thus laid a solid foundation for the development of organoid technology. A breakthrough came in 1975 with the work of Rheinwald and Green, who found that co‐cultures of primary human keratin‐forming cells and 3T3 fibroblasts were able to form stratified squamous epithelial colonies similar to those of human epidermis, which provided an important reference for the mimicry of organoids in skin tissue development [34]. Organoid research advanced further into the 1980s when researchers successfully isolated pluripotent stem cells from mouse embryos and demonstrated that these cells had the ability to differentiate into one or more of the desired mature cell types, thus possessing the potential to repair organs [35]. This discovery opened up new avenues for the use of organoids in disease modeling and regenerative medicine. In 1987, researchers began to explore methods of culturing primary cells on tumor‐reconstituted basement membranes to simulate more complex 3D culture systems. Then, in 2009, Hans et al. achieved another milestone in the field of organoid research. They successfully used adult intestinal stem cells to culture intestinal organoids, which were able to self‐assemble and differentiate into intestinal tissues with crypt villi [36, 37]. The method was subsequently successfully used for the construction of stomach [38], pancreas [39], colon [37], liver [40], brain [41], and bone [42] organs. This achievement not only demonstrates the potential of organoid technology in modeling complex organ structures but also lays a solid foundation for subsequent organoid applications.

## 3 Periosteum Organoid

Periosteum organoid, as an important area in the application of organoid technology, aims to reproduce the complex 3D structure organization and functional natural properties of periosteum in vitro [43]. By precisely regulating the proliferation, differentiation and interaction of stem cells on the 3D scaffold material, and at the same time simulating the biochemical signals and mechanical stimuli in the periosteum microenvironment in vivo, periosteum organoids with high physiological similarity can be constructed [44, 45]. The periosteum organoid not only provides a powerful tool to study the biological processes of bone development, fracture healing and bone regeneration, but also lays a solid foundation for the development of novel bone repair strategies.

### 3.1 Periosteum

The periosteum, a key component of the skeletal system, covers almost most of the outer surface of the bone, and it acts as a bridge and transition zone between the bone and the adjacent soft tissues [46]. This layer of structure is not only highly functional and complex, but also plays a crucial role in both physiologic and pathologic processes of the skeleton.

From an anatomical point of view, the periosteum can be meticulously divided into two main layers: the fibrous layer and the cambium layer [47, 48]. There is no clear boundary between these two layers, but this can be seen histologically especially during osteoid apposition and together they form a fully functional system. The cambium layer is in direct contact with the bone surface, and it is enriched with osteogenic stem and progenitor cells. These cells are key players in the bone healing and regeneration process, and they provide the cellular support necessary for bone repair and reconstruction [49]. The fibrous layer, on the other hand, serves as the nutrient supply layer within the bone and contains fibroblasts, collagen, and a rich network of capillaries. These structures not only provide the periosteum with the necessary nutrients and oxygen, but also help to maintain the normal physiological function of the periosteum. In addition, some of the fibers in the fibrous layer penetrate the bone to form the so‐called “Sharpeys fibers,” which act as anchors between the periosteum and shaft and also with ligaments and tendons, further enhancing the stability and integrity of the bone structure [50, 51].

The role of the periosteum is even more important in the process of bone repair and reconstruction. Whether it is the reconstruction of bone defects caused by skeletal diseases or the healing of fractures, the periosteum is able to provide necessary support and assistance for bone repair and regeneration through its internal cellular components and fiber structure. Therefore, the study and understanding of periosteum not only helps us to better understand the physiological and pathological processes of periosteum in promoting bone repair, but also provides new ideas and methods for the treatment of skeletal diseases and bone repair techniques in the future.

### 3.2 Design of Periosteum Organoid

In the design of periosteum organoids, the construction strategy is systematically divided into two core elements: basic design and functional design [52]. As the cornerstone of organoid construction, basic design provides a strong material foundation and technical support for the construction of organoids by integrating key elements such as cells, scaffolds, growth factors, and microfluidic systems (Table 1) [53, 54]. Functional design, on the other hand, explores and expands the physiological functions of periosteum in bone repair, and optimizes and promotes the process of bone repair through the comprehensive effects of antimicrobial, immunomodulation, neuromodulation, angiogenesis, and osteogenesis. This process not only reflects the cross‐disciplinary integration of biomaterials science, cell biology, and tissue engineering, but also opens up new horizons and possibilities for research and clinical application in the field of bone regenerative medicine.

#### 3.2.1 Basic Design

The basic components of a periosteum organoid (and more broadly, organoid construction in tissue engineering) include key aspects such as cells, scaffolds, growth factors, and microfluidic systems. The role of each of these elements in the construction of periosteum organoids is considered below.

##### 3.2.1.1 Cells

Cells are the basic unit for building organoids and occupy a central position in regenerative engineering. In this complex and delicate process, researchers prefer to use cell populations with significant osteogenic potential, such as MSCs and periosteal stem cells. These cells can show multidirectional differentiation ability, and can not only differentiate into osteoblasts to participate in the formation and mineralization of bone matrix, but also transform into chondrocytes or adipocytes according to the environmental signals, thus playing a crucial role in the construction and functional recovery of periosteal tissues [55].

##### 3.2.1.2 Scaffold

The selection of scaffold materials is particularly important in the complex construction of periosteum organoids. Scaffolds not only provide a solid support structure for cell growth, but also need to have appropriate porosity to support cell migration, proliferation, and secretion and deposition of extracellular matrix in 3D space [56]. Commonly used scaffold materials include natural polymers, synthetic polymers, and inorganic materials. Ideal scaffold materials should combine biocompatibility, degradability, and appropriate mechanical strength and space to provide an ideal environment for cell growth, thereby promoting regeneration and functional recovery of periosteal tissues.

##### 3.2.1.3 Growth Factors

Introducing and regulating the type and concentration of growth factors is a key component in inducing the directed differentiation of stem cells into osteoblasts, thereby accelerating bone tissue formation and repair [57, 58]. For example, bone morphogenetic proteins (BMPs), transforming growth factor‐β (TGF‐β), insulin‐like growth factor (IGF), platelet‐derived growth factor (PDGF), vascular endothelial growth factor (VEGF), fibroblast growth factor (FGF), tumor necrosis factor‐α (TNF‐α), as typical osteogenic growth factors, have been proved to have strong osteogenic potential, and they act as mediators of intercellular signaling, which can accurately regulate the rate of proliferation, direction of differentiation, and expression of specific functional proteins in stem cells [59, 60]. Therefore, the strategy of adding growth factors can not only accurately guide the differentiation of stem cells toward osteogenesis, but also optimize the construction process of periosteum organoid and promote the rapid formation and efficient repair of bone tissue.

##### 3.2.1.4 Microfluidic System

Microfluidics technology utilizes the creation of a unique network of microchannels and microvalve design to achieve fine manipulation of fluid flow paths and flow rates [61]. This highly adjustable fluid management system ensures that the cells are able to obtain an appropriate amount of nutrients and oxygen uniformly and continuously to meet their metabolic needs for growth and differentiation. At the same time, metabolic waste is removed in a timely manner, avoiding the accumulation of harmful substances that can adversely affect cell health [62]. This technology promotes the precise regulation of cell growth and differentiation, but also opens up a new way for in‐depth exploration of biological developmental mechanisms, construction of disease models, and development of organoid strategies.

**TABLE 1:** Basic design of periosteum organoid.

Element	Type	Function	Ref.
Cells	BMSCs	One of the most important osteogenic differentiated stem cell types.	[63]
	Periosteum‐derived cells (PDC)	Possesses clonal pluripotency and self‐renewal capacity, mainly intramembranous osteogenesis.	[64]
	Skeletal stem cells	Mainly endochondral osteogenesis.	[65]
	Macrophage‐lineage tartrate‐resistant acid phosphatase–positive (TRAP^+^) cells	Secretion of PDGF‐BB recruits PDC to the periosteal surface and differentiates them into osteoblasts.	[66]
Scaffold	Extracellular matrix	The decellularized extracellular matrix is a naturally cross‐linked and highly ordered 3D fibrillar network providing a model for in vivo/in vitro mineralization.	[67]
	Collagen fiber	Prevents invasion of reticular fibrous tissue and promotes osteogenic mineralization.	[68]
	Piezoelectric poly(vinylidene fluoride‐trifluoroethylene) (PVFT)	Repairing the local osteogenic electrical microenvironment to induce stem cell migration.	[69]
	Poly(lactic‐co‐glycolic acid) (PLGA), polycaprolactone (PCL), poly(l‐lactic acid) (PLLA)	Excellent biocompatibility and biodegradability, which significantly promote osteogenic differentiation and meet the required mechanical properties of bone.	[70, 71]
	Gelatine, hyaluronic acid, chitosan, poly(oxyethylene) (PEG)	As a classic material for hydrogels, it can provide a 3D skeleton for bone repairs.	[72, 73, 74, 75]
Growth factors	BMPs, TGF‐β	Regulation of osteogenesis‐related gene expression through activation of the SMAD signaling pathway.	[76, 77]
	IGF	Promotion of osteoblast proliferation, differentiation and mineralization through the PI3K/Akt signaling pathway.	[78, 79, 80]
	PDGF, VEGF	Bone regeneration by promoting angiogenesis.	[81]
	TNF‐α	Often associated with inflammatory responses and can indirectly regulate osteogenesis through activation of RANK.	[82]
	FGF	Promote osteoblast proliferation and differentiation	[83]
Microfluidic system	Linear channel	Single fluid flow for basic cell culture or drug screening.	[84, 85]
	Networked channel	Simulation of complex fluid dynamics, for example, simulation of vascular networks or complex periosteal blood flow.	[86, 87]

#### 3.2.2 Functional Design

The periosteum has antimicrobial, immunomodulatory, neuromodulatory, angiogenic, and osteogenic functions that protect damaged bone and ensure effective bone repair. It is important to design periosteum organoids that accurately mimic all the complex functions of the periosteum for regeneration, appropriate for both open and closed fractures, and the nature of periosteal organoids with different functions is now considered.

##### 3.2.2.1 Antimicrobial

Periosteum acts as a barrier membrane that prevents invasion of surrounding connective tissue and bacterial infection in open injuries, providing a favorable microenvironment for osteogenesis [88]. A bacterial infection of the tissue is considered among the most devastating complications of surgery [89]. It is often caused by the invasion of fungal or bacterial pathogens into the interior of the wound, and severe bone infections can progress further to osteomyelitis [90]. The progression of bone infection can be broadly divided into three stages [91]: Pathogen entry into host cells to evade antibiotics and immune responses: For example, *Staphylococcus aureus* anchors with osteoblasts through fibronectin binding protein, fibronectin, and integrin a5b1 [92]. It is also the main pathway of pathogen invasion into host cells. Biofilm formation: When *S. aureus* adsorbs to the substrate, the increase in extracellular polymeric material leads to further aggregation of *S. aureus* together to form biofilms [93]. Biofilms prevent entry of antibiotics and immune cells, further increasing the difficulty of treatment [94]. Bone destruction: On the one hand, *S. aureus* entry into cells induces expression of TRAIL to reduce osteoblast activity and trigger bone destruction. On the other hand, bioactive factors interact and stimulate osteoclasts, leading to bone loss and destruction [95, 96]. Reports in the literature suggest that there is great variability in the incidence of bone infections, ranging from 1%–55%, depending on the type of surgery, patient lifestyle, and other chronic conditions [97, 98, 99]. Bone infections without therapeutic intervention contribute to bone healing failure, repeated surgeries, and increased treatment costs. Designing periosteum organoids with antimicrobial properties can play a crucial role in achieving successful osteogenesis. Strong support for bone tissue repair and regeneration can be accomplished by integrating antimicrobial mechanisms and biomaterial technologies.

##### 3.2.2.2 Immunomodulation

The inflammatory response is the first stage in initiating the bone healing process. Multiple macrophage subpopulations exist in natural periosteum, and functional transitions between different macrophage subpopulations are thought to be a key mechanism for coordinating bone repair and healing. As a critical cell type for osteoimmunity, macrophages can differentiate into different subtypes under the influence of the microenvironment, which participate in the regulation of bone metabolism. M1 macrophages secrete pro‐inflammatory cytokines, which differentiate into osteoclasts and enhance bone resorption, whereas M2 macrophages secrete anti‐inflammatory factors, which stimulate osteoblast differentiation, thereby promoting bone formation [100]. Once macrophage polarization is out of balance, the healing cascade is disrupted, leading to delayed or even no healing. In addition, researchers have found that by using immunohistochemistry, inflammatory macrophages such as the F4/80^+^Mac‐2^hi^ population are activated in the periosteum after injury [101]. This finding not only confirms the central role of osteoimmunity in the bone healing phase but also suggests that specific interactions of the immune system are equally crucial during bone repair. These interactions include signaling between immune cells and osteoblasts, the release of immunomodulatory molecules, and the shaping of the immune microenvironment, which together form a complex regulatory network that finely regulates every stage of bone healing. Therefore, it is important to investigate in depth the biological mechanisms of different subpopulations of periosteal macrophages during bone formation and bone resorption for bone healing.

##### 3.2.2.3 Neuromodulation

Recently, the regulation of bone regeneration by nerves has received widespread attention. During embryonic development, it has been found that nerve tissue develops earlier than bone tissue, which may be related to the fact that the brain develops earlier than bone in the embryo [102]. Clarifying the regulatory role of nerves for bone has great implications for bone tissue engineering. The periosteum is characterized by an abundant neural network, which regulates bone formation and bone resorption through neural signals to maintain bone homeostasis together [103, 104]. Notably, the periosteum is not only densely populated with sensory neurons but is also rich in sympathetic neurons, which finely regulate bone metabolic processes by releasing a series of biologically active factors such as neuropeptides and cytokines [105, 106]. In addition, the regulation of bone metabolism by the nervous system involves more far‐reaching dimensions. Studies have shown that the central nervous system is not only directly involved in the maintenance of skeletal homeostasis but also indirectly affects bone metabolism by regulating the secretion of leptin, a key metabolic hormone [107, 108, 109]. As a “messenger” between the nervous system and the skeletal system, leptin plays an important role in promoting bone formation and inhibiting bone resorption. When the interconnection between the nervous system and the skeletal system is disturbed, bone homeostasis is disrupted, and various diseases such as bone tumors and heterotopic ossification occur [110]. Consequently, although bone homeostasis is linked to the activity of the nervous system, there is still limited research on the communication mechanisms between the two systems, and further in‐depth studies are required.

##### 3.2.2.4 Angiogenesis

An abundance of blood vessels in the periosteum can transport the required nutrients, oxygen, minerals, and metabolic wastes, which are necessary for the bone healing process [111]. Neovascularization, that is, the formation and development of new blood vessels, forms an integral part of the bone repair mechanism as well as the construction of periosteum organoids. When bone tissue is injured, it will first form a hematoma, and immune cells are recruited. Then fibroblasts and chondrocytes form healing tissue with the help of neovascularization, and the healing tissue is further mineralized and deposited to complete bone repair [112, 113]. VEGF is a key factor in vascularized osteogenesis. In mouse experiments, inhibition of VEGF function negatively affected bone formation, again demonstrating the importance of vascularization for success [114, 115]. Vascularity is essential for all bone healing and therefore, must be considered in design strategies for bone tissue engineering. The most challenging issue in vascularized bone tissue engineering is how to connect the new microcirculatory vessels to the organism, as the connection between these vessels themselves is not an immediate process may take up to 8 days [116, 117]. Therefore, the importance of vascularization during bone repair cannot be overstated. It is not only related to the speed and quality of healing, but also a key factor in determining the success of periosteum organoid construction. In the future, how to guide the blood vessels to be rapidly neovascularized and seamlessly integrated with the organism will become a hotspot and a challenge in the field of bone tissue engineering, and also an important direction to promote technological breakthroughs in this field.

##### 3.2.2.5 Osteogenesis

The periosteum is not only directly involved in osteogenesis but also indirectly catalyzes the advancement of the osteogenic process through multiple mechanisms, such as nutrient supply and growth factor release. The periosteum contains a large number of cells with osteogenic potential and plays an extremely important role in the process of osteogenesis by providing the substances and cells necessary for the osteogenic phase [118, 119]. Osteoblast differentiation is regulated by a variety of factors, including physical, chemical, and biological stimuli [120]. There are two main types of repair osteogenesis involving the periosteum: direct bone healing, known as intramembranous ossification, and indirect cartilage formation followed by remodeling to mature bone, known as endochondral ossification. The process of intramembranous ossification is relatively simple. When the periosteum contacts the hematoma site, the periosteal stem cells are affected by microenvironmental factors, differentiate into osteoblasts, and calcify to form mature bone tissue [121, 122]. Endochondral ossification is the main form of osteogenesis after periosteal implantation [123]. The process begins with the formation of the hematoma and its stabilization with the surrounding soft tissue, followed by the active differentiation of stem cells within the cartilage and the production of chondrocytes in large numbers. Over time, this cartilaginous healing tissue is gradually replaced and evolves into mature bone tissue [124, 125]. Furthermore, a study has shown that periosteum‐derived stem cells exhibit greater growth potential and osteogenic differentiation capacity than BMSC [65]. This finding further highlights the importance of local periosteal response mechanisms and activation of periosteal stem cells in the region of injury during the process of bone repair, which play an indispensable role in promoting bone regeneration and recovery.

## 4 Application of Periosteum Organoid

### 4.1 Antibacterial Periosteum Organoid

**FIGURE 3:**
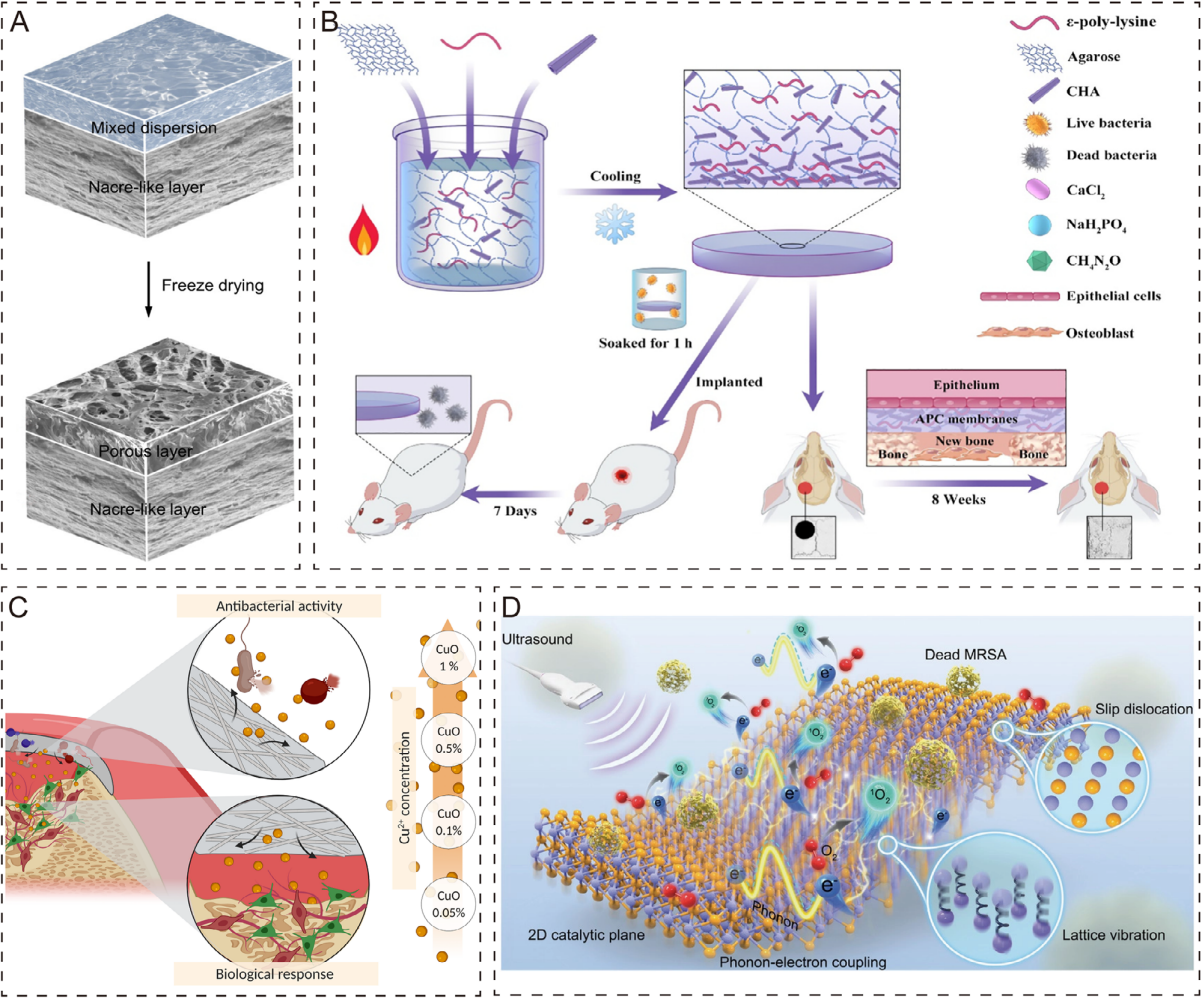
Periosteum organoid with antimicrobial function. (A) Design of bilayer biofilms with high mechanical properties and effective bacterial inhibition by evaporation‐induced self‐assembly and ice template technology [128]. Copyright 2019, Elsevier Inc. (B) ε‐PLL, as an antimicrobial agent, showed excellent antimicrobial effects during osteogenesis [135]. Copyright 2021, Elsevier Ltd. (C) Cu^2+^‐loaded resorbable electrospun membranes prevent bacterial infections while promoting bone resorption [145]. Copyright 2022, Elsevier B.V. (D) Ultrasonic stimulation triggers a coupling effect between phonons and surface electrons to treat MRSA‐induced bone infections with a 99.72% sterilization rate [148]. Copyright 2023, Wiley‐VCH GmbH.

Periosteum organoids with antimicrobial properties are highly desirable. Bacterial infections frequently accompanying the implantation process of conventional bone substitutes are a serious problem in the bone healing process that is difficult to ignore [126]. In clinical treatment, systemic antibiotic therapy is usually used, but high doses of antibiotics can cause host and bacterial resistance and even more serious infections [127]. To solve the above problems, a periosteum organoid biomaterial with antibacterial function would be a good solution. Chitosan and graphene oxide have received widespread attention as commonly used antimicrobial biomaterials that can be prepared as bilayer guided bone regeneration (GBR) membranes with synergistic osteogenic and antimicrobial functions to repair bone defects (Figure 3A) [128]. As commonly used antimicrobial drugs in clinical practice, antibiotics such as tetracycline, ciprofloxacin, and tobramycin have demonstrated excellent efficacy [129, 130, 131]. However, with the emergence of bacterial resistance and multidrug‐resistant bacteria, new alternatives are urgently needed to meet this challenge. In this context, novel antimicrobial materials with novel antimicrobial properties, such as AgNPs [132], chitosan‐based biomaterials [133], bacterial peptides and antimicrobial alloys [134], have shown promising development prospects. Researchers have added ε‐polylysine (ε‐PLL) to agarose hydrogels, which not only have favorable antimicrobial properties but also support osteogenic cell development (Figure 3B) [135]. Metallic elements are essential for both biological functions and the metabolism of cells. Silver (Ag) [136, 137], gold (Au) [138, 139], copper (Cu) [140], zinc (Zn) [141], and their corresponding oxides have received much attention in bacteriostatic studies [142, 143, 144]. In a recent study, Cu^2+^ was loaded into electrospun membranes, and it was found that adjusting the concentration of Cu optimized antimicrobial activity and exhibited excellent antimicrobial properties (Figure 3C) [145]. In addition to the antibiotics, antimicrobial drugs, polymers, and inorganic metals mentioned above, antimicrobial properties can also be achieved by external stimuli (photocatalysis and sonocatalysis). The advantages of using the latter methods are rapid and efficient sterilization with no side effects. Despite exciting advances in photocatalysis for antimicrobial purposes, therapeutic effects on bone tissue are limited by the limited penetration [146]. In attempts to address this issue, sonocatalysis methods are emerging as attractive alternatives. Ultrasound, as a mechanical wave, has superior tissue penetration, thus providing a more concentrated antimicrobial effect on the infected area with no significant attenuation of energy [147]. Mao et al. designed a novel acoustic sensitizer that can stimulate electron transfer via ultrasound and promote O_2_ production, thereby effectively treating MRSA‐induced infections and reducing the inflammatory response of bone tissue (Figure 3D) [148].

Periosteum organoids with antimicrobial function are gradually showing their important value, which can effectively prevent and treat bone infections and promote bone defect repair, and also significantly reduce postoperative complications, bringing new hope for bone regenerative medicine. However, the high cost of these materials and the complex preparation and processing technologies have become the main obstacles limiting their wide application. Future research should focus on three major aspects:

1. To develop new antimicrobial composites, balance antimicrobial and biocompatibility, and explore cost‐effective preparation processes.
2. The promotion of intelligent design by the use of smart materials to accurately regulate the antimicrobial properties.
3. To focus on personalization to achieve the delivery of precision medicine to patients.

### 4.2 Immunomodulatory Periosteum Organoid

**FIGURE 4:**
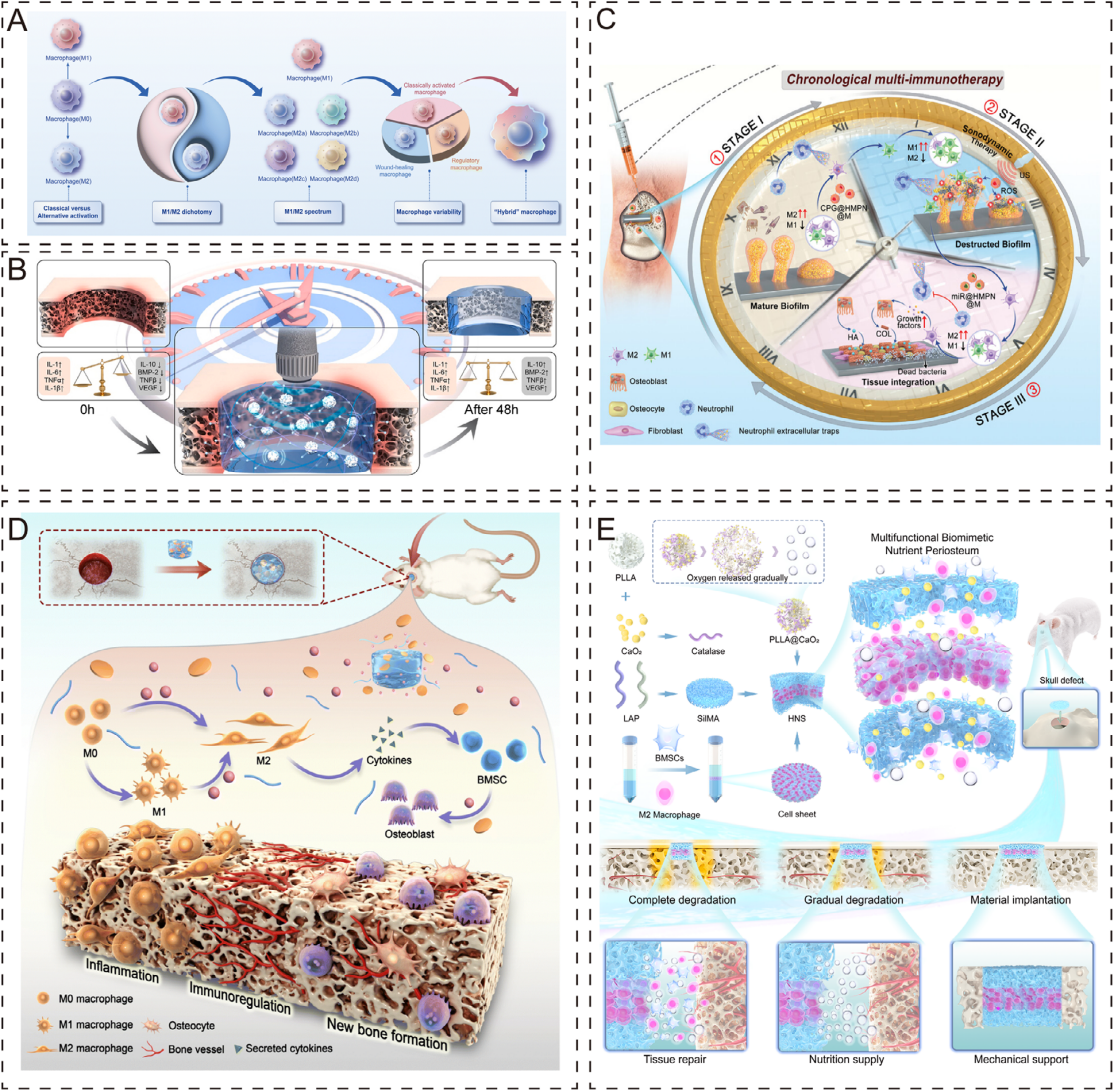
Periosteum organoid with immunomodulatory function. (A) Regulation of immunity by different subtypes of macrophages [151]. Copyright 2023, The Royal Society of Chemistry. (B) Composite hydrogel combined with ultrasound stimulation for rapid release of anti‐inflammatory agents [152]. Copyright 2023, Elsevier Ltd. (C) Novel nano‐targeted adjuvants enable time‐sequenced immunotherapy and promote macrophage polarization to M1 and M2 in a sequential manner [153]. Copyright 2023, Wiley‐VCH. (D) Complex hydrogel can effectively regulate the polarization ratio of macrophages (inhibit M1, promote M2), and promote bone regeneration [154]. Copyright 2023, The Royal Society of Chemistry. (E) Three layers of periosteum organoid combined with cell sheet technology significantly modulate the immune response to promote bone repair [63]. Copyright 2024, Elsevier B.V.

The immune system and bone repair are closely related, sharing many cytokines and other signaling molecules (Figure 4A) [149, 150, 151]. Engineering periosteum organoids adapted to the bone immune microenvironment contributes to bone repair and osseointegration. Acute inflammation usually occurs within 48 h after bone injury. Han et al. designed a novel immunomodulatory composite hydrogel, which, combined with ultrasonic stimulation, can continuously release anti‐inflammatory drugs with a drug release rate of 38.14%, achieving rapid treatment (Figure 4B) [152]. Some researchers have argued that since inflammation in tissue repair is a dynamic process, therapeutic strategies that focus only on a specific time are insufficient to meet therapeutic needs. Wang et al. proposed that customized treatments in chronological order were effective in improving inflammation and promoting tissue repair [153]. This immunomodulatory material achieves the functions of blocking pathogens and activating inflammation, macrophage polarization to M1‐type elimination of biofilm, and macrophage repolarization to M2‐type inhibition of inflammation and promotion of tissue repair in a chronological order (Figure 4C). In another study, Li et al. also achieved targeted polarization of macrophages with the help of biomaterials to regulate immune function [154]. The researchers added the immunobioactive agent harmine to the hydrogel, and through in‐depth analysis by transcriptome sequencing, found that the hydrogel activated M2 macrophages, upregulated anti‐inflammatory cytokines (IL‐4, IL‐10, and Arg‐1), and activated the PI3k‐Akt signaling pathway to promote osteogenesis (Figure 4D). In order to simulate the immune microenvironment of periosteum better, Hao et al. designed a hamburger‐like periosteum containing BMSCs and M2‐type macrophages, which could effectively regulate the polarization of macrophages to the M2 phenotype and achieve effective regulation of bone immunity (Figure 4E) [63]. In summary, it is clear that the polarized state of macrophages and the beneficial immune microenvironment have important regulatory roles in bone regeneration [155].

Periosteum organoids with immunomodulatory functions can significantly enhance their biocompatibility, reduce inflammation, and create a conducive environment for bone regeneration. Among them, it is also crucial to deeply investigate the specific interactions between the immune system and periosteum organoids. This interaction is not only related to the ability of periosteum organoids to play a stable immunoregulatory role in a complex and variable biological environment, but also directly affects their efficiency and effectiveness in promoting bone regeneration. By precisely analyzing the signaling mechanism between immune cells and periosteum organoids, we can better understand the biological basis for their promotion of bone healing, and thus provide a scientific basis for optimizing the design of periosteum organoids, so that they can be more accurately matched to the individual patient's needs, and the therapeutic efficacy of periosteum organoids can be maximized.

### 4.3 Neuromodulatory Periosteum Organoid

The nervous system serves as an early participant in bone repair, with both the central and peripheral nervous systems influencing skeletal developmental events [156]. More specifically, nerve growth factor (NGF) deficiency results in damaged innervation and decreased osteocytes. The neural factors include serotonin (5‐HT), calcitonin gene‐related peptide (CGRP), substance P (SP), and acetylcholine (Ach) [157], which interact with receptors on osteocytes (Table 2). Based on this, the design of biomaterial scaffolds/membranes carrying nerve cells and growth factors plays a crucial role in the bone regeneration process. According to the recent study, researchers inoculated BMSC on PCL nanofiber nerve guide conduits and co‐cultured them. The results showed a significant increase in nerve synapses in rats, which could promote peripheral nerve regeneration in the injury gap [158]. In addition, extracellular exosomes and extracellular vesicles, as important components of tissue engineering, have been rapidly developed in regenerative medicine due to their safety and efficacy [159]. Hao et al. verified the important role of innervation of bone regeneration by exosomes derived from Schwann cells [160]. Su et al. used phosphatidylserine (PS)‐targeted aptamers coupled with exosomes from Schwann cells loaded onto the surface of electrospun fibers to construct an artificial bionic periosteum [161]. Both in vivo/in vitro experiments demonstrated that this neuromodulated bionic periosteum has significant neurogenesis and also promotes angiogenesis and osteogenesis.

**TABLE 2:** Connections between the nervous system and the skeletal system.

Nervous system	Bioactive factor	Receptor	Function
Central nervous system	5‐HT	5‐HT receptors	Peripheral 5‐HT inhibits mouse osteoblast proliferation and differentiation and promotes bone resorption [162].
	Semaphorin 3 A	Neuropilin‐1 (Nrp‐1)	Sema 3A activates the Wnt/β‐catenin signaling pathway and inhibits adipocytosis and osteoclastogenesis [163].
	Brain‐derived neurotrophic factor (BDNF)	Tyrosine Kinase receptor B (TrkB)	BDNF activates ERK1/2 and AKT signaling pathways and promotes the proliferation and differentiation of hMSCs [164].
Peripheral nervous system	CGRP	RAMP1‐CLR complex	Activation of the cAMP‐CREB pathway promotes osteogenic differentiation of MSCs [165].
			CGRP inhibits RANKL‐NF‐κB signaling pathway to suppress osteoclast differentiation [166].
	SP	NK1‐R	High concentrations of SP stimulated the up‐regulation of osteogenic gene expression in BMSC [167].
	Vasoactive intestinal peptide (VIP)	VPAC1 and VPAC2 receptors.	VIP activation of the Wnt/β‐catenin pathway enhances differentiation of BMSC [168].
	Neuropeptide Y (NPY)	Y1 and Y2 receptors	NPY promotes the proliferation and differentiation of BMSC into osteoblasts via the Wnt/β‐catenin pathway [169].
			NPY combined with Y2 receptors modulates noradrenergic neurons and attenuates excessive stress‐induced bone loss [170].
	NE	β‐adrenergic receptors (β‐AR)	NE promotes osteoclast maturation [171].
	Ach	nAChRs and mAChRs	ACh stimulates osteoblast proliferation [172].
			Activation of nAChR inhibits RANKL‐induced Ca^2+^ production, leading to osteoclastogenesis [173].

In conclusion, periosteum organoids with neuromodulation offer an innovative and highly promising therapeutic strategy for bone repair. However, the development of this field is still in its infancy, and there are still many issues to overcome. For example, how to explore the mechanism of interaction between neurotransmitters and osteoblasts, and how to construct a microenvironment for dynamic nerve‐bone balance. Further research should focus on key scientific questions with a view to promoting breakthroughs in neuromodulation technology in the field of bone repair, and ultimately achieving safer, more efficient, and personalized treatment protocols for bone injuries.

### 4.4 Angiogenesis Periosteum Organoid

**FIGURE 5:**
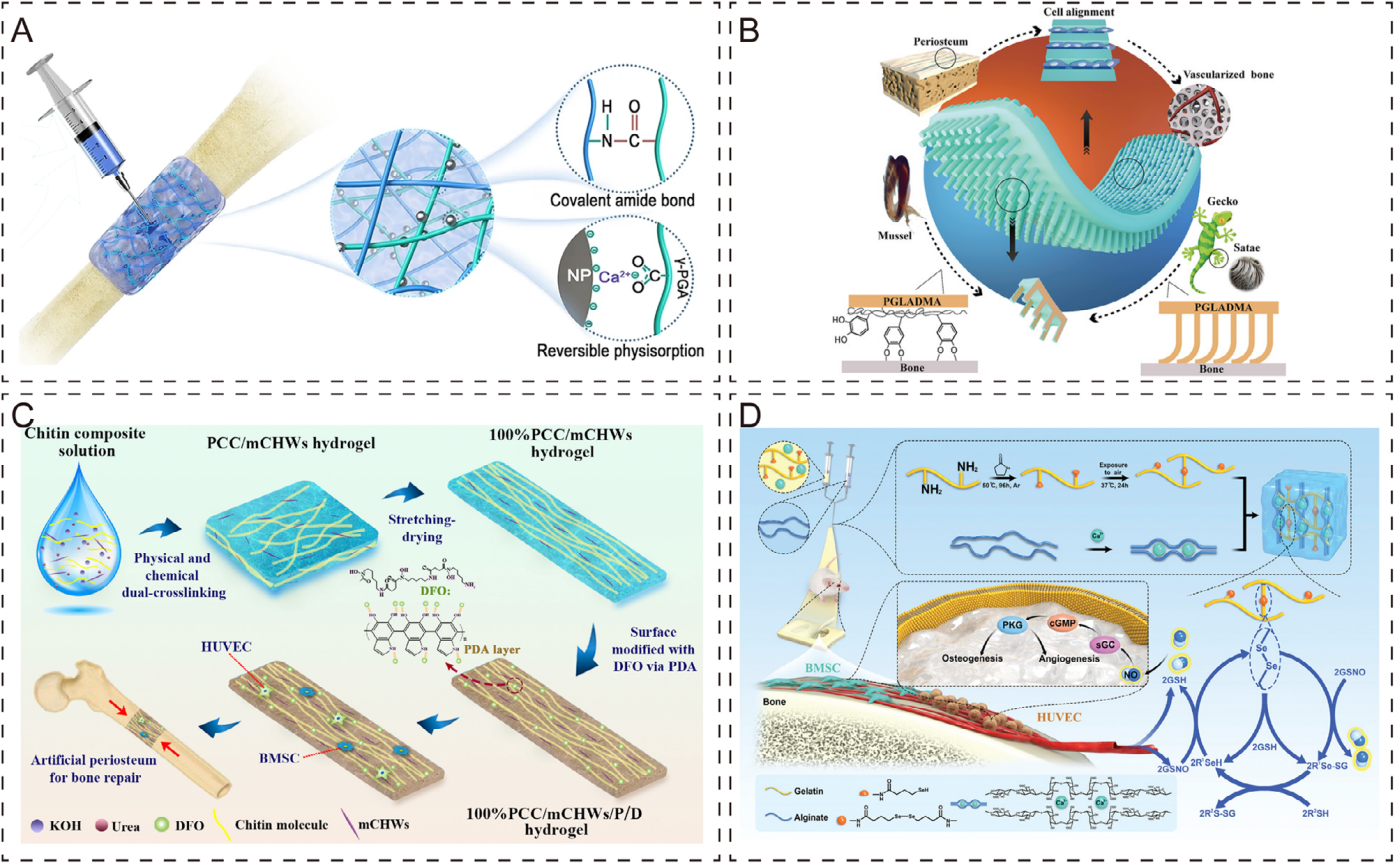
Periosteum organoid with angiogenesis function. (A) Dual cross‐linked composite hydrogel promotes vascularized bone regeneration by recruiting BMSC and endothelial cells [177]. Copyright 2022, BioMed Central Ltd. (B) Gecko‐inspired strongly adhesive Janus artifacts with vascularized properties [179]. Copyright 2021, Wiley‐VCH GmbH. (C) Anisotropic composite hydrogels loaded with DFO for enhanced osteogenic and angiogenic activity [180]. Copyright 2022, Elsevier Ltd. (D) Adaptive periosteum organoid that continuously releases NO activates vasculogenic signaling pathways coupling osteogenesis and angiogenesis [194]. Copyright 2023, Wiley‐VCH GmbH.

As mentioned above, natural periosteum contains a rich vascular network that provides oxygen and nutrients to the skeletal system. Therefore, it is very important to induce vascular neovascularization in tissue engineering and construct a vascular network to obtain a more functional periosteum organoid. Currently, design strategies for periosteal vascularization revolve around scaffold design, seed cells, growth factors, signaling cues, and pre‐vascularization [174]. Hydrogel, as a hydrophilic 3D mesh structure with an internal structure similar to that of natural periosteum, has very obvious advantages in constructing biomimetic periosteum [175, 176]. For example, Lou et al. designed a composite hydrogel inspired by periosteum using PEGylated poly (glycerol sebacate) polymer and CaP nanoparticles, which exhibited significantly enhanced angiogenic and osteogenic activities by virtue of its excellent adhesion and bioactivity (Figure 5A) [177]. For the design strategy of the periosteum organoid, the researchers not only studied the natural structure of the periosteum in depth, but also referred to other structures in nature to enhance the versatility of the periosteum organoid. Conventional design strategies for artificial periosteum focus less on tissue adhesion, leading to inward growth of soft tissue, thus creating scar tissue that hinders the osteogenic process [178]. Inspired by the gecko, the researchers designed fibrillar setae arrays incorporating mussel adhesion proteins to enhance the adhesion of the periosteum organoid under dry/wet conditions. Experimental results showed that this Janus periosteum also has significant advantages in osteogenesis and vascularization (Figure 5B) [179]. He et al. also improved the design of the hydrogel by adding a hypoxia‐mimicking compound that accelerates vascular regeneration (Figure 5C) [180]. The direct addition of seed cells (MSC [181, 182], endothelial cells [183]), growth factors (VEGF [184], bFGF [185], PDGF [186], HIF‐1α [187]), metal ions (B, Mg, Sr, Cu, Co, Ti, and Zn) [188, 189, 190, 191], and nitric oxide (NO) [192, 193] cues when designing the periosteum organoid is also an effective treatment strategy. For example, NO can act as a bioactive agent that activates the NO‐cyclic guanosine monophosphate (cGMP) signaling pathway, which coordinates the vascularized osteogenesis process and promotes bone repair (Figure 5D) [194]. The release pattern of signaling cues is also critical. In addition to the common uniform release, release modes such as combined release, delayed release, and programmed release need to be designed for different disease conditions. This could be an influential future research direction for vascularized bone tissue engineering. Other than the above‐mentioned approaches, pre‐vascularization techniques are equally important. Pre‐vascularization refers to the pre‐generation of a scaffold with a vascular network in vivo or in vitro so that the scaffold can provide a rich blood supply immediately after implantation and quickly establish a connection with the body [195, 196]. Recently, lithography techniques and 3D printing have provided entirely new ways to fabricate microchannels for capillaries and blood vessels [197, 198, 199].

Due to the critical role of adequate blood supply in the process of bone repair to ensure the structural and functional regeneration of bone tissues, the construction of an efficient and stable blood supply network has become an indispensable part of the field of bone regenerative medicine. Although the stability of these methods still needs to be improved, they have shown encouraging results in enhancing the efficient delivery of oxygen and nutrients to the repair site, and this progress opens up new avenues for the advancement of periosteum organoids. In this process, the importance of vascularization is particularly prominent. Vascularization is not only about the adequacy of the blood supply, but also about bridging the new bone tissue to the body's pre‐existing vascular system, which is an indispensable physiological process during bone repair. Pre‐vascularization techniques, in particular, are considered to be highly promising due to their ability to significantly facilitate rapid angiogenesis in the clinical setting. However, due to the complexity and technical difficulty of its construction process, more in‐depth exploration and research is still needed by researchers. In the future, with the continuous advancement of technology and innovation of materials, we have reason to believe that pre‐vascularization technology will play an even more important role in the field of bone repair, and provide more solid support for the clinical application of periosteum organoids.

### 4.5 Osteogenesis Periosteum Organoid

**FIGURE 6:**
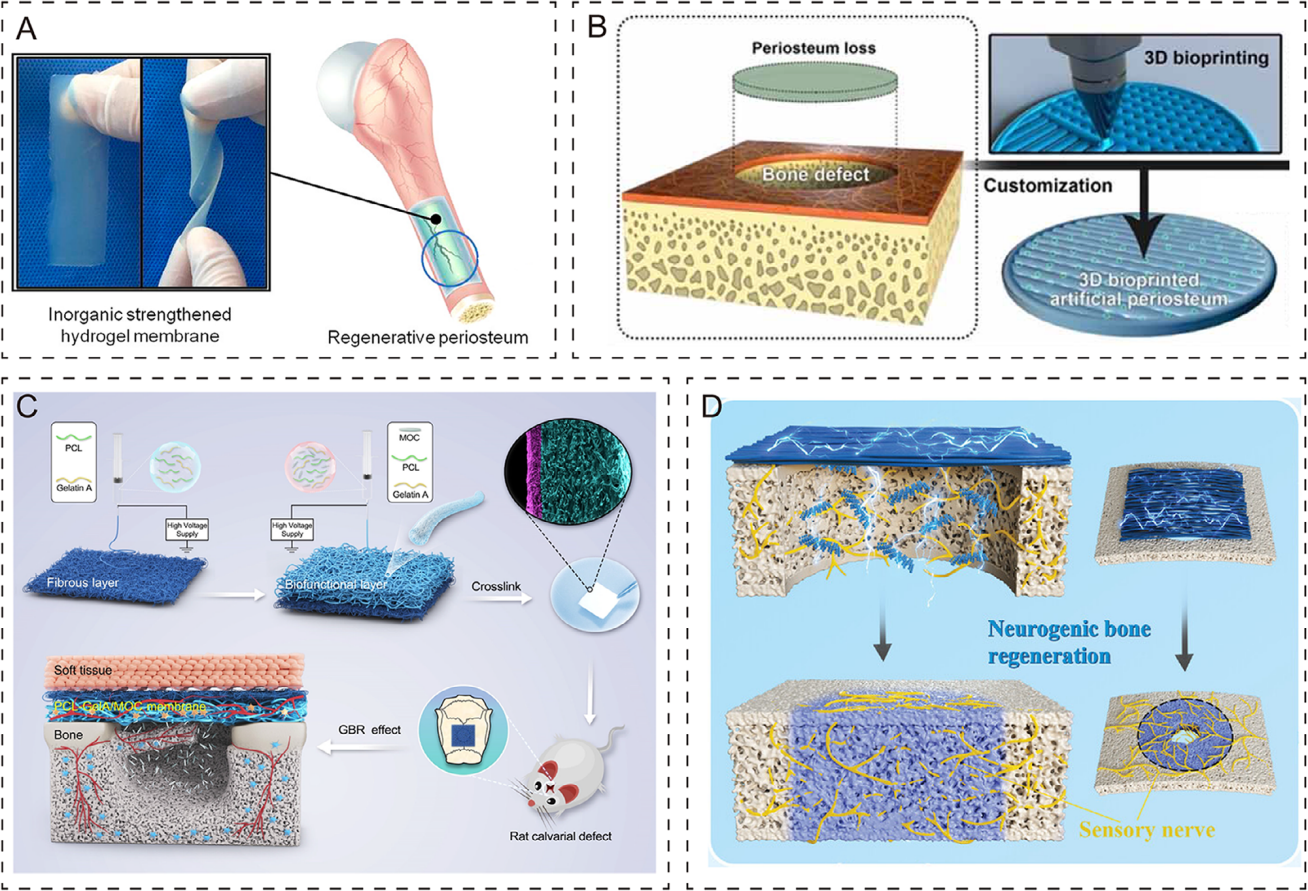
Periosteum organoid with osteogenesis function. (A) Organic/inorganic co‐crosslinked hydrogel membranes enhance bone regeneration in rat cranial defects [204]. Copyright 2017, American Chemical Society. (B) 3D printing of graphene oxide‐loaded periosteum organoid promotes osteogenic differentiation and accelerates bone regeneration in BMSC [207]. Copyright 2023, IOP Publishing Ltd. (C) Double‐layer electrospun membrane loaded with osteoinductive factors promotes vascular regeneration to assist bone regeneration [211]. Copyright 2022, American Chemical Society. (D) Endogenous electric field‐coupled bionic periosteum promotes nerve‐induced bone regeneration [214]. Copyright 2023, Wiley‐VCH GmbH.

The core function of the periosteum organoid is to have excellent osteogenic potential, which accelerates and optimizes the repair process of bone defects. Currently, construction strategies for periosteum organoids are divided into scaffold‐based and scaffold‐free approaches. Scaffolds templates for initial cell attachment, migration, proliferation, and differentiation, and are capable of manipulating cellular morphology to direct the orderly synthesis of biological processes [200, 201]. Hydrogel, as a network scaffold similar to the ECM environment, becomes an ideal choice for tissue engineering [202, 203]. Xin et al. designed a hydrogel membrane formed by co‐cross‐linking bioactive glass and gelatin derivatives, and implanted it into rat cranial defects to observe its repair effect [204]. The results showed that this hydrogel membrane has excellent angiogenic and osteogenic abilities (Figure 6A). With the development of bone tissue engineering, 3D printing technology allows for precise design at macro and micro scales, providing a more suitable in vivo response for periosteum organoid. The physicochemical properties (rheology, compression strength, adhesion, degradation) and cellular activity of different bioinks affect the restorative effect, making the selection of the right bioink crucial [205, 206]. Sun et al. selected graphene oxide/gelatin/silk proteins/dopamine‐modified gelatin composite bioink to 3D print a novel periosteum organoid (Figure 6B) [207]. Electrostatic spinning, a technique for spray spinning polymer solutions in a strong electric field, has received increasing attention due to its wide range of flexibility, effectiveness, and unique physicochemical properties [208, 209, 210]. Zhu et al. prepared a fiber layer with osteoinductive function using a mixed solution of polycaprolactone‐gelatin‐magnesium oxychloride ceramics [211]. Magnesium oxychloride ceramics act as osteoinductive factors, delivering chemical signals and recruiting stem cells to provide the proper osteogenic microenvironment for bone reconstruction (Figure 6C). In addition, it has been shown that bone tissue also promotes bone repair when electrically and mechanically stimulated [212, 213]. Recently, a black phosphorus electrostatically spun bionic periosteum was prepared, and the activation of the bionic periosteum under an endogenous electric field released sensory neurotransmitters and promoted the differentiation of BMSC into osteoblasts (Figure 6D) [214]. The promotion effect of electrical stimulation on bone repair was verified. Li et al. then used collagen to fabricate natural bone‐periosteal scaffolds, resulting in stable bone‐periosteal structures [215]. Unlike biomaterial scaffolds, decellularized matrix and cell sheet technology can effectively avoid problems such as scaffold degradation and immune rejection. As an alternative approach, cell sheet technology is mainly maintained by cell‐cell junctions and secretion of ECM proteins, and the intact cell sheet structure results in excellent cell activity and regeneration [216, 217, 218]. Using MSC cell sheets as tissue‐engineered periosteum to wrap allografts, Long et al. observed that the cell sheets formed cartilage at the host junction, promoted bone scab formation, and observed large amounts of periosteal healing tissue [219, 220].

When exploring the application of periosteum organoids for the promotion of bone defect repair, it is not difficult to find that these biomaterials, whether or not relying on scaffolding structures, exhibit certain technical limitations. These limitations significantly hinder their potential to flexibly respond to the complex and changing demands of bone healing in clinical practice. Bone healing is a highly complex and synergistic multi‐system process involving osteoblasts, blood vessels, growth factors, immunity, and biomechanics. Of particular note, osteogenic function, as a core aspect of bone healing, is critical for achieving effective repair of bone defects. However, the current efficacy of periosteum organoids in inducing osteogenesis is still insufficient to fully meet the urgent need for efficient and precise bone regeneration in clinical practice. In summary, future research should focus on breaking through the limitations of existing technologies and developing a class of periosteum organoids with highly integrated functionality through innovative design, with the aim of realizing more accurate, efficient, and comprehensive bone defect repair in clinical practice.

## 5 Summary and Prospect

In conclusion, periosteum occupies a central position in the process of bone repair and regeneration, and its importance cannot be ignored. The periosteum not only carries a rich reserve of osteogenic‐related cells, but is also a major source of supply of growth factors, which are essential for the formation of new bone and the repair of old bone. Therefore, the periosteum is regarded as a key player in the mechanism of bone regeneration. The high degree of synergy between the immune, vascular, nervous, and skeletal systems is particularly critical in the process of periosteal promotion of bone repair. The immune system supports bone repair by regulating inflammatory and immune responses; the vascular system provides the periosteum with essential nutrients and oxygen while removing metabolic wastes; the nervous system influences the bone repair process through neurotransmitters and hormones; and the skeletal system is directly involved in bone formation and remodeling. This close cooperation between multiple systems ensures the efficiency and accuracy of the periosteum in bone repair.

In view of the importance of periosteum in the bone healing process, the focus of this paper is on the design of periosteum organoids. We summarize a series of periosteum organoids with different functional properties, aiming to mimic and optimize the natural process of bone regeneration (Table 3). These periosteum organoids not only have the ability to generate and differentiate osteoblasts, but also secrete various growth factors to support bone repair. They also have the potential to interact with the immune, vascular, and nervous systems to better integrate and facilitate the overall process of bone repair. Specifically, the antimicrobial function can protect the repair site from infection and create a sterile environment for tissue regeneration. Immunomodulation helps to balance the local immune response, reduce the negative impact of inflammation on bone healing, and promote the formation of a microenvironment conducive to tissue repair. Neuromodulation is essential for restoring sensory and motor functions at the site of injury, which promotes the growth and connection of nerve fibers and accelerates the full recovery of functions. Angiogenesis ensures adequate blood supply to the new bone tissue, which is essential for maintaining cellular activity and promoting tissue maturation. Osteogenic function is the core of periosteum organoid material, which directly determines the efficiency and quality of bone defect repair. Different functional periosteum organoids have unique strengths, and the limitations of any single strategy may result in a significant reduction in overall efficacy. In view of this, future tissue engineering research needs to urgently turn to the development of a periosteum organoid material that is multifunctional in nature. Such materials should not only be able to induce osteogenic activity, but also have the ability to respond to dynamic changes in the microenvironment. On this basis, further integration of antimicrobial, immunomodulatory, neuromodulatory, and other multidimensional functions will greatly enrich the clinical potential of periosteum organoid materials.

**TABLE 3:** Strategies for periosteum organoid design.

Type	Aim	Limitation	Ref.
Antibacterial periosteum organoid	Prevents bacterial infection, promotes integration of the materials into the tissue and accelerates the tissue regeneration and healing process.	Inadequate control of drug release.	[126, 221]
		Inadequate broad‐spectrum antimicrobial capacity.	
Immunomodulatory periosteum organoid	Regulates the immune response, improves the immune microenvironment, and prevents excessive immune response leading to tissue damage or dysfunction.	Lack of in‐depth understanding of mechanisms.	[149, 222]
		Limited material responsiveness	
		Poor long‐term stability.	
Neuromodulatory periosteum organoid	Integration of the central nervous system allows the periosteum organoid to sense and respond to external stimuli, thereby more accurately mimicking the physiological function of the periosteum.	Complex neuromodulatory mechanisms.	[223, 224, 225]
		Limited neuro‐skeletal synergy.	
		Challenging clinical applications.	
Angiogenesis periosteum organoid	Provides essential nutrients and oxygen supply to bone tissue while transporting growth factors and other bioactive molecules to promote repair and regeneration of bone tissue.	Long generation time of the vascular network.	[226, 227]
		Poor long‐term stability.	
Osteogenesis periosteum organoid	The main physiological functions of the periosteum, promoting osteoblast differentiation, proliferation, and bone matrix synthesis etc.	Limited time‐space regulation of osteogenic factors.	[228]
		Lack of long‐term follow‐up studies.	

Periosteum organoids, as a complex and unique organoid system in biology, have shown great potential in the field of bone repair and regenerative medicine. However, in order to realize their full potential for clinical applications, future intense exploration of the specific mechanisms of action in bone repair, combined with their further optimization and design, is critical.

1. In‐depth exploration of the mechanisms of action: Periosteum organoids play multiple roles in the process of bone repair, including providing cell sources, releasing growth factors, and promoting blood vessel formation. Future research should focus on the detailed analysis of these mechanisms, with a view to enhancing the effect of bone repair through human intervention and optimization.
2. Multi‐functional integration: With the continuous progress of biotechnology, periosteum organoids should develop in the direction of multi‐functional integration. This means that we need to focus on the basic functions of promoting bone repair, together with investigations on the potential applications for drug delivery, immunomodulation, tissue engineering, and all aspects of functional tissue regeneration. By integrating multiple functions, periosteum organoids are expected to become an important tool for bone repair and regenerative medicine in the future.
3. Feasibility assessment: In order to ensure the scientific validity and effectiveness of periosteum organoid design, we need to conduct a rigorous feasibility assessment. This includes the design, implementation, and analysis of in vivo/in vitro experiments, as well as focusing on evaluating their performance and stability under different conditions. Through these assessments, we can continuously optimize the design and improve the effectiveness of periosteum organoids in bone repair.
4. Optimization of compatibility of independent organoid structures: As an independent organoid system, periosteum organoids need to be highly compatible and synergistic with other biological tissues. Therefore, we need to focus on the interface with other biological tissues and investigate methods to ensure the function of periosteum organoids while improving compatibility with the surrounding tissues. By optimizing compatibility, we can further improve the effectiveness of periosteum organoids in bone repair and reduce possible complications.

In summary, periosteum plays a crucial role in the process of bone repair, and the design and application of periosteum organoids provide new ideas and methods for bone repair. With increasing research activity and the rapid advancement of organoid technology, there is no doubt that even more innovative and practically useful periosteum organoids will be developed in the future, bringing renewed hope and novel possibilities to the field of bone repair.

## Conflicts of Interest

The authors declare no conflicts of interest.
